# The Relationship Between Undulatory Underwater Kick Performance Determinants and Underwater Velocity in Competitive Swimmers: A Systematic Review

**DOI:** 10.1186/s40798-022-00485-0

**Published:** 2022-07-28

**Authors:** Rani West, Anna Lorimer, Simon Pearson, Justin W. L. Keogh

**Affiliations:** 1 Faculty of Health Sciences and Medicine, Bond University, Gold Coast, QLD Australia; 2 Sports Performance Research Centre New Zealand, AUT University, Auckland, New Zealand; 3 Queensland Academy of Sport, Nathan, QLD Australia; 4 Cluster for Health Improvement, Faculty of Science, Health, Education and Engineering, University of the Sunshine Coast, Sippy Downs, Australia; 5 Kasturba Medical College, Mangalore, Manipal Academy of Higher Education, Manipal, Karnataka India

**Keywords:** Swimming, Performance, Undulatory underwater swimming, Biomechanics, Kinematics

## Abstract

**Background:**

Undulatory underwater swimming (UUS) has become an integral component of the start and turn phases in competitive swimming allowing higher velocities than can be achieved swimming at the surface. An understanding of the most important determinants for UUS performance and how these can be optimised to different swimmers is poorly understood.

**Objective:**

The aim of this systematic review was to systematically assess the current peer-reviewed literature on the relationship between UUS performance determinants and underwater velocity in competitive swimmers.

**Methods:**

An electronic search using AusSportMed, Embase, PubMed, SPORTDiscus and Biomechanics and Medicine in Swimming was performed. The methodological quality of the studies was evaluated using a biomechanics-specific checklist developed by Hindle and colleagues (Sports Med Open. 5(1):49, 2019. 10.1186/s40798-019-0222-z).

**Results:**

Twenty-five studies met the eligibility criteria. While UUS velocity was nearly perfectly related (*r* > 0.90) to foot resultant acceleration and kick frequency, several other biomechanical factors were also significant correlates. UUS velocity and frequency were typically higher in high-performance swimmers and during prone versus dorsal positions. UUS velocity, kick frequency and kick amplitude were also significantly correlated with high angular velocities of the hip, knee and ankle joints and knee range of motion.

**Conclusion:**

While there appears to be evidence supporting some performance variables to be related to UUS, future research should examine how to optimise the kinematic and kinetic characteristics with respect to the imposed task constraints and organism constraints between swimmers. Additional research should also investigate the effect of biomechanically informed interventions to improve UUS performance.

***Registration*:**

Open Science Framework.

## Key Points

- A range of kinematic and kinanthropometric parameters are strongly correlated with undulatory underwater velocity; however, foot resultant acceleration, kick frequency, kick amplitude, vertical toe velocity and knee angular velocity appear to be the greatest predictors of high UUS velocity.
- Swimmers should perform the glide at approximately 0.4 m underwater at all velocities above 1.9 ms-1 to gain maximum drag reduction benefits, where a 15-18 % reduction in underwater total drag was found when compared to swimming at the surface.
- An athlete’s optimal movement combination when performing undulatory underwater swimming may be different to others, as each swimmer needs to exploit their own organism constraints to maximise propulsive impulse while simultaneously reducing drag in response to the task and environmental constraints.

## Introduction/Background

Pool swimming is a foundation Olympic sport incorporating four recognised strokes: freestyle, butterfly, backstroke and breaststroke, with events ranging from 50 to 1500 m [2, 3]. Competitive pool swimming events can be divided into four distinct phases; the start, free swimming, turn and finish. The phases can be defined as: the start (time to 15 m), free swimming (parts of the race not including start, turn or finish), turn (5 m into the wall and 10 m out) and finish (5 m into the wall on the last lap). With early swimming research focused primarily on the free swimming component, more recent studies have recognised the contribution of the other phases of the race as important determinants for overall performance [4, 5]. Undulatory underwater swimming (UUS) has become an integral component of the start and turn phases since the 1980 Olympic Games in Moscow, where swimmers began to prolong the underwater phase, applying undulatory swimming to minimise the loss of velocity until the initiation of the above water stroke [6, 7]. The timing and types of UUS are also key factors for minimising deceleration from initial velocity from the dive or wall start [8]. Specifically, the undulatory kick is preferable to flutter kicking as it reduces deceleration before the initiation of the free swimming portion [8]. International Swimming Federation (FINA) regulations currently allow a swimmer to remain underwater for 15 m during the start and turn phases of the race [9], meaning the UUS may contribute up to 30% of race distance during a standard long course event [10]. Performance determinants of the UUS can be broadly categorised as ways to minimise resistance drag and/or to improve propulsive force production.

The total drag forces experienced by the swimmer reflect wave, skin and profile drag [11]. Because the density of water is approximately 800 times higher than that of air, the drag force in water is also higher [8]. The UUS promotes improved propulsive efficiency compared to swimming at the surface by eliminating wave drag [12, 13]. As swimming velocity, frontal surface area and frontal shape are the primary determinants of form drag force, the influence of frontal area and shape can be reduced by adopting a streamlined, horizontal position, with both arms overstretched and held together in front of the head while performing UUS [14–16]

Human underwater undulatory motion is dependent on the swimmer’s ability to produce propulsive forces, which are primarily generated by a wave running cephalocaudally along the athlete’s body [17]. The undulatory wave increases in amplitude at each subsequent body segment in a whip-like action, where momentum is transferred from larger body segments to smaller ones [10, 18, 19] and to the water resulting in a propulsive impulse [20]. Underwater velocity is equal to product of kick frequency and horizontal distance per kick, and the optimal interaction between these two factors [16, 21]. The cyclic vertical motion of the lower limbs caused by the body wave has the greatest amplitude at the toes, which is related to vortex creation and thrust production [10, 15, 19]. It has been stated that body wave velocity and vertical toe velocity could potentially explain differences in UUS performance, with implications for improving athlete technique [15].

An athlete’s ability to optimise propulsion and minimise resistance in the UUS may also be influenced by their anthropometry, range of motion and flexibility. The tendency for swimmers to be tall and lean assists in reducing drag, with their long limbs contributing to greater stroke length and/or kick amplitude [22]. However, there are some anthropometric differences between male and female swimmers and those who specialise in different strokes and distances [22]. A swimmer’s anthropometry may also have conflicting effects on their movement efficiency. When performing the UUS, the increase in displacement from the cranial to caudal body segments is not smooth due to the limited number of rotational joints available in the human body, compared to aquatic animals [23]. Range of motion and flexibility may then play an important role in creating and maintaining the optimal body position during UUS by allowing the swimmer to reduce resistance forces and/or increase propulsive force production [17].

Despite the frequent use of the UUS in training and competition, the factors determining its effectiveness in high-performance swimmers remain somewhat unclear [24]. This relative lack of understanding may partially reflect the most recent review on UUS published by Connaboy et al. [25] that only included six studies and the major changes that have occurred in the dive start since that time. Specifically, high-performance swimmers now use a kick or track start technique on the OSB11 start block (OMEGA, Zurich, Switzerland), which was first introduced in 2010. It provides a number of biomechanical and performance advantages for swimmers during the starting phase [26–28]. The purpose of this systematic review was to systematically assess the current peer-reviewed literature on the relationship between UUS kinematic, kinetic and kinanthropometric (anthropometric and physical fitness) factors that may influence UUS velocity in swimmers.

## Methods

### Experimental Approach to the Problem

A review protocol for this paper was developed using the Preferred Reporting Items for Systematic Reviews and Meta-Analyses’ (PRISMA) guidelines on reporting items for a systematic review and the associated PRISMA checklist [29]. The protocol was registered with Open Science Framework (https://osf.io/), and a set of inclusion and exclusion criteria were developed prior to undertaking the search process, as summarised in Table 1.

**Table 1:** Inclusion and exclusion criteria for the systematic review

Inclusion		Exclusion
*General*		
Article type	Full peer-reviewed journal article Conference articles that provided sufficient detail regarding study methodology and results	Recommendation articles Review articles (non-original work) Editorials Magazine articles Computational Studies/Numerical Investigations Abstracts/Summaries/Not full article Includes study of animals Articles that cannot be found
Date	No restrictions	
Language	English only	Languages other than English
*Participants*		
Age	Mean age of 16 and above	Mean age of under 16 years old
Sex	Male and/or Female participants	
Level	Human competitive swimmers of a national OR international OR Olympic level Regional- or state-level swimmers were included if data was separate from national and international swimmers	Untrained, novice, masters and Paralympic swimmers. Aquatic athletes from sports other than swimming, including water polo and triathlon
Health	Swimmers currently training and competing	Studies of post injury biomechanics/rehabilitation studies
*Study protocol*		
Outcomes	Articles including outcomes related to underwater dolphin kick Kinematics Kinetics Kinanthropometry Undulatory underwater kick performed in a prone or dorsal position	

### Search Strategy

A structured literature search, using established search terms appropriate for each of the following databases: PubMed, SPORTDiscus, Embase and AusSportMed, was initially carried out with assistance from the University Faculty librarian on the 23 January 2020 and repeated on the 4 May 2021. A search of the database International Symposium on Biomechanics and Medicine in Swimming was then completed on the 6 December 2021. The search strategy included Medical Subject Headings (MeSH) terms and key words related to the primary concepts of the overall research question, using the PICO (population, intervention, comparison/control and outcome) approach. [The full search used included: swim* AND (dolphin OR undulat* OR underwater) AND kick* as the basis for searches in the other databases. For PubMed: (swim* OR "Swimming"[Mesh]) AND (dolphin OR undulat* OR underwater) AND kick*; for SPORTDiscus: (swim* OR DE "SWIMMING" OR DE "INDIVIDUAL medley" OR DE "LONG distance swimming" OR DE "MEDLEY relay (Swimming)" OR DE "MIDDLE distance swimming" OR DE "SWIMMING competitions" OR DE "SWIMMING for people with disabilities" OR DE "SWIMMING for women") AND (dolphin OR undulat* OR underwater) AND kick*; for Embase: (swim* OR 'swimming'/exp) AND (dolphin OR undulat* OR underwater) AND kick*; and for AusSportMed: swim* AND (dolphin OR undulat* OR underwater) AND kick*].

### Risk of Bias and Quality Assessment

A risk of bias and quality assessment was undertaken by two independent reviewers. As no standard checklist appeared to be entirely suitable for eligible cross-sectional biomechanical studies in this review, a checklist developed by Hindle and colleagues [1] and subsequently used by several other authors was utilised [1, 30, 31]. When any disagreements in the scoring between reviewers occurred, a consensus meeting was held to establish an agreement. An item was scored as one if the article provided sufficient evidence in support of the criteria, and zero where the criteria were not met. A total risk of bias score was calculated for each article and categorised using the methods of Davids and Roman [32], with articles scoring ≥ 67% considered as having a low risk of bias, articles scoring in the range of 34–66% considered as having a satisfactory risk of bias and articles scoring ≤ 33% considered as having a high risk of bias.

### Data Extraction and Analysis

Database results were combined, and duplicates were deleted. Titles and abstracts were screened for key words, and citations were then categorised into inclusion and exclusion sets to determine total number of records for synthesis. Data were extracted before risk of bias assessment was conducted using Endnote as the reference management software package. Dual screening was then carried out, using all inclusion and exclusion criteria by two independent reviewers, by titles and abstracts. For articles that appeared to meet the inclusion criteria, or where it was not clear, full-text reports were examined. The same two reviewers then independently screened the full text against the eligibility criteria, and any discrepancies were discussed with an experienced third researcher to reach a consensus. Reasons for any exclusions were recorded.

Data extracted from each eligible study included descriptive information of the study population, including number, age, performance level (regional club, state, national and international) and stroke identified. Data regarding the study design, UUS parameters measured, outcome(s) measured, statistical relationships and main findings reported were also extracted. For studies that reported correlations between the reported outcomes, qualitative descriptions of strength of the correlations were provided based on Hopkins using the following criteria: < 0.1, trivial; 0.1–0.3, small; 0.3–0.5, moderate; 0.5–0.7, large; 0.7–0.9, very large; > 0.9, nearly perfect [4].

## Results

### Study Characteristics, Methodology and Review Statistics

**Fig. 1:**
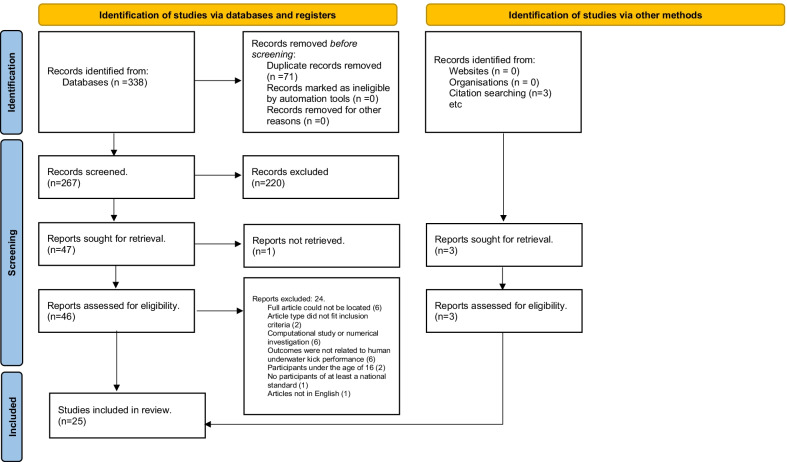
PRISMA flow chart [29]

Figure 1 represents the article review process based on the Preferred Reporting Items for Systematic Reviews and Meta-Analyses (PRISMA) guidelines [29]. The literature search identified 338 studies. Of the 46 articles retained for full-text screening, 22 articles were identified as being adherent to the inclusion criteria and three additional studies were identified through other sources, yielding a total of 25 studies included in the systematic review. The results of the search process are illustrated in a PRISMA flow chart in Fig. 1.

### Risk of Bias and Quality Assessment

All studies clearly stated the objectives or purpose of the study, described the testing methods and results and had a study design that adequately tested the hypothesis. The majority of studies clearly described the characteristics of the population and provided sufficient information that would allow the reader to make an unbiased assessment. All articles were classified as having a satisfactory or low risk of bias (Table 2) [1].

**Table 2:** Method for assessing quality and risk of bias

Article	1.1	1.2	1.3	2.1	2.2	2.3	2.4	3.1	3.2	3.3	3.4	4.1	4.2	4.3	4.4	4.5	Score (%)
Alves et al. [6]	0	1	1	0	1	0	0	1	0	1	1	1	1	1	0	0	56.3 (S)
Arellano et al. [33]	0	1	1	0	1	0	0	1	0	1	1	1	0	1	0	0	50 (S)
Atkison et al. [10]	0	1	1	0	1	1	0	1	1	1	1	1	1	1	0	1	75 (L)
Connaboy et al. [34]	0	1	1	1	1	1	0	1	0	1	1	1	1	1	1	1	81.3 (L)
de Jesus et al. [35]	0	1	1	0	1	0	0	1	0	1	1	1	0	1	0	0	50 (S)
Elipot et al. [14]	0	1	1	0	1	0	0	1	0	1	1	1	1	1	0	0	56.3 (S)
Higgs et al. [15]	0	1	1	0	1	1	0	1	1	1	1	1	1	1	1	1	81.3 (L)
Hochstein and Blickhan, [7]	0	1	1	0	0	0	0	1	0	0	1	1	1	1	1	0	50 (S)
Hochstein and Blickhan [17]	0	1	1	0	1	0	0	1	0	1	1	1	1	1	1	0	62.5 (S)
Hochstein et al. [44]	1	1	1	0	0	0	0	1	1	0	0	1	1	1	0	0	50 (S)
Houel et al. [36]	1	1	1	0	1	0	0	1	1	0	1	1	1	1	1	1	75 (L)
Houel et al. [37]	1	1	1	0	1	1	0	1	0	1	1	1	1	1	1	1	81.3 (L)
Ikeda et al. [41]	0	1	1	0	1	0	0	1	1	1	1	1	1	1	1	1	75 (L)
Jensen and McIlain [45]	1	1	1	0	1	0	0	1	0	0	0	1	1	1	1	0	56.3 (S)
Lyttle et al. [43]	0	1	1	1	1	0	0	1	1	1	1	1	1	1	0	0	68.8 (L)
Lyttle and Blanksby [42]	0	1	1	1	1	0	0	1	0	0	0	1	1	1	0	0	50 (S)
Miwa et al., [46]	1	1	1	0	0	0	0	1	0	0	0	1	1	1	1	0	50 (S)
Shimojo et al. [47]	0	1	1	1	1	1	0	1	0	0	0	1	1	1	0	0	56.3 (S)
Shimojo et al. [16]	0	1	1	1	1	0	0	1	0	1	1	1	1	1	1	1	75 (L)
Shimojo et al. [48]	1	1	1	1	1	1	0	1	0	1	1	1	1	1	1	1	87.5 (L)
Shimojo et al. [38]	1	1	1	0	1	0	0	1	0	0	0	1	1	1	1	1	62.5 (S)
Wang and Liu [39]	0	1	1	0	1	1	0	1	0	1	1	1	1	1	0	0	62.5 (S)
Willems et al. [24]	0	1	1	1	1	1	0	1	0	1	1	1	1	1	0	1	75 (L)
Yamakawa et al. [40]	0	1	1	0	1	0	0	1	0	1	1	1	1	1	0	1	62.5 (S)
Yamakawa et al. [49]	1	1	1	0	1	0	0	1	1	1	1	1	1	1	0	0	68.8 (L)
Total criteria met/25	8	25	25	7	22	8	0	25	7	17	19	25	23	25	12	11	-

(1.1) study design is clearly stated; (1.2) the objectives/purpose of the study are clearly defined; (1.3) the design of the study adequately tests the hypothesis; (2.1) the criteria for the inclusion of subjects are clearly described; (2.2) the characteristics of the population are clearly described; (2.3) the study sample is representative of the population intended to the study; (2.4) a description of how the study size was arrived at is provided; (3.1) the testing methods are clearly described; (3.2) the measurement tools used are valid and reliable; (3.3) the statistical methods used are well described; (3.4) the statistical tests used to analyse the data are appropriate; (4.1) the results are well described; (4.2) the information provided in the paper is sufficient to allow a reader to make an unbiased assessment of the findings of the study; (4.3) confounding factors are identified; (4.4) sponsorships/conflicts of interest are acknowledged; and (4.5) any limitations to the study are identified. Note: the risk of bias score for an article (given as a percentage) is calculated through the addition of the score from each criteria being met divided by the maximum possible score across all criteria (16), multiplied by 100. L low risk of bias (67–100%), S satisfactory risk of bias (34–66%), H high risk of bias

### Study Results and Data Synthesis

Table 3 summarises the demographic characteristics of the participants as well as the primary methodological characteristics of the study, number of trials and the biomechanical analysis used in each study. The sample sizes of the included articles ranged from 1 to 40 participants, with mean ages between 16 and 26 years. Studies used various methodologies to test their hypotheses, with 1 to 3 trials of 10–20 m maximum UUS performance efforts the most common approach assessed [6, 7, 10, 14–17, 24, 33–44]. Nineteen of the 25 included studies utilised 2D kinematic analyses [6, 7, 10, 14–17, 24, 33, 34, 37, 39–43, 45–47] with 8 of these studies collecting data of how active kinanthropometric measures such as joint range of motion may be related to UUS performance [6, 10, 15, 17, 33–35, 41]. Of the 6 studies that performed a kinetic analysis, 4 studies calculated net and drag force data and reported the coefficient of drag [42–45], and 2 used a swimming flume to examine flow characteristics during UUS [38, 46].

**Table 3:** Summary of study participants, demographic characteristics, trials performed, kinematic, kinetic and kinanthropometric analysis

References	Participants	Demographic characteristics (mean ± SD)	Trials performed	Kinematic analysis	Kinetic analysis	Kinanthropometric analysis
Alves et al. [6]	6 junior national swimmers^a^	17.0 ± 0.4 yrs 177.0 ± 3.6 cm 69.3 ± 6.0 kg	3 × 25 m max UUS from a block start in dorsal, prone and lateral positions	Two fixed underwater digital cameras (front and sagittal) with a frame rate of 50 Hz. Images from the 4th kick cycle were retained for 3D kinematic analysis		Active ankle and knee range of motion
Arellano et al. [33]	11 national swimmers (M)	19.9 ± 2.2 yrs 184.7 ± 5.8 cm 75.7 ± 8.7 kg	2 × 15 m max UUS with an underwater start in prone and dorsal positions	One underwater sagittal camera with a frame rate of 50 Hz		Active full body range of motion
Atkison et al. [10]	15 adult international and state swimmers (M)	21.5 ± 3.2 yrs	3 × 15 m max UUS from a push start in prone body position	One underwater sagittal camera with a frame rate of 30 Hz		Active full body range of motion
Connaboy et al. [34]	17 national swimmers (8 M, 9F)	Male swimmers: 17.6 ± 1.4 yrs 177.6 ± 5.3 cm 72.7 ± 7.9 kg Female swimmers: 16.4 ± 0.8 yrs 164.9 ± 4.1 cm 53.8 ± 3 kg	3 × 15 m max UUS from a push start in prone body position	One underwater sagittal camera with a frame rate of 50 Hz		Active full body range of motion
de Jesus et al. [35]	4 international swimmers	22.8 ± 1.7 yrs 178.0 ± 6 cm 76 ± 8.9 kg	2 x (3 × 15 m) max UUS in dorsal body position	A cable velocimeter with a sampling rate of 50 Hz		Active lower body range of motion
Elipot et al. [14]	12 national swimmers (M)	183 ± 5 cm 76.1 ± 5.2 kg	3 × 15 m max UUS from a grab start in prone body position	Four underwater mini-DV camcorders with a frame rate of 25 Hz		
Higgs et al. [15]	7 national swimmers (7 M, 3F)	21.1 ± 2.6 183.0 ± 8 cm 79.5 ± 10.1 kg	3 × 20 m max UUS from a push start in prone body position	One underwater sagittal camera with a frame rate of 100 Hz		Active lower body range of motion
Hochstein and Blickhan [7]	2 national swimmers (F)	Subject 1: 26 yrs 178.0 cm 73 kg Subject 2: 24 yrs 167 cm 56.5 kg	15 m max UUS trials from a standing start in prone body position	Two underwater sagittal cameras (one on each side). One camera was used for motion capture and the other was used for flow capture		
Hochstein and Blickhan [17]	4 national swimmers (F) 6 regional club swimmers (3 M, 3F)	22.1 ± 4.3 yrs 171.4 ± 5.9 cm 65.4 ± 9.4 kg	10 m max UUS trials from a standing start in the prone body position	One underwater sagittal camera with a frame rate of 125 Hz		Active full body range of motion
Hochstein et al. [44]	1 national swimmer (F)	NA	20 m max UUS trial from a standing start in the prone body position	One underwater sagittal camera with a frame rate of 250 Hz	2D Particle Image Velocimetry (PIV)	Active full body range of motion 3D Body Scanner
Houel et al. [36]	12 national swimmers^a^	21.41 ± 4.5 yrs 183.33 ± 4.9 cm 75.8 ± 5.1 kg	10 m max UUS trials from a grab start in prone body position	Three underwater cameras (two in the sagittal plane, one recording a slanting view of the swimmer motion) with a frame rate of 50 Hz 3D kinematic analysis		
Houel et al. [37]	10 national swimmers^a^	21.41 ± 4.5 yrs 183.33 ± 4.9 cm 75.8 ± 5.1 kg	10 m max UUS trials from a grab start in prone body position	Three underwater cameras (two in the sagittal plane, one recording a slanting view of the swimmer motion) with a frame rate of 25 Hz 3D kinematic analysis		
Ikeda et al. [41]	9 swimmers (M) FINA 766 ± 91.4	20.4 ± 1.67 yrs 174 ± 0.06 cm 69.5 ± 6.73 kg	3–5 × 15 m max UUS trials in prone body position	One underwater camera in the sagittal plane with a frame rate of 120 Hz and exposure time 1/500 s		Active full body range of motion
Jensen and McIlain [45]	2 international swimmers (1 M, 1F)	NA		One underwater sagittal camera with a frame rate of 48 Hz	Segmental size parameters were calculated and used with the mean densities by Clauser et al. (1969) to give the inertial parameters needed for kinetic analysis	Anthropometric measurements taken of the lower extremity of the swimmers were used to formulate a geometric representation of the segments
Lyttle et al. [42]	Study 1 40 national swimmers (M)	NA	Towed in a prone position 25 m at depths 0.6, 0.4 and 0.2 m underwater and at the surface. At each depth, swimmers were towed at 1.6 to 3.1 m s^−1^ in 0.3 m s^−1^ increments	One underwater sagittal camera. Frame rate not stated A variable-control, motorised winch and pulley system to accurately and consistently maintain a set velocity	Unidirectional load cell (frame rate not provided)	
	Study 2 16 national swimmers (M)		Towed 25 m at a depth of 0.5 m underwater at velocities 1.6; 1.9; 2.2; 2.5 and 3.1 m s^−1^. At each velocity the swimmer performed maximal prone and lateral streamline glide, prone freestyle kick and, prone and lateral undulatory kick			
Lyttle et al. [43]	16 national swimmers	19.3 ± 2.1 yrs 181 ± 5 cm 77.8 ± 6.2 kg	Towed 25 m at a depth of 0.5 m underwater at velocities 1.6; 1.9; 2.2; 2.5 and 3.1 m s^−1^. At each velocity the swimmer performed maximal prone and lateral streamline glide, prone freestyle kick and, prone and lateral undulatory kick	One underwater sagittal camera. Frame rate not stated A variable-control, motorised winch and pulley system to accurately and consistently maintain a set velocity	Unidirectional load cell (frame rate not provided)	
Miwa et al. [46]	1 national swimmer (M)	NA	5 × steady UUS in a swimming flume (1.0 m s^−1^)	One underwater sagittal camera with a frame rate of 15 Hz	Nylon tracer particles (50 μm) were admixed to the flume. A Nd:YAG laser was placed below the flume and illuminated the flow area in a sagittal plane	
Shimojo et al. [47]	15 national swimmers (10 M, 5F)	22.1 ± 4.7 yrs	Task 1 10 × 10 m UUS trials from a push start in the prone body position at different kick frequencies using a target sound	Four underwater sagittal view cameras with a sampling frequency of 60 Hz 2D analysis Target sounds at 75% (375–825 Hz) and 50% (450–750 Hz) kick frequency generated by underwater speakers		
			Task 2 10 × 10 m UUS trials from a push start in the prone body position with no target sound	Four underwater sagittal view cameras with a sampling frequency of 60 Hz		
Shimojo et al. [16]	10 national swimmers (M)	21.3 ± 0.9 yrs 175.5 ± 5.4 cm 71.3 ± 4.8 kg	15 m maximal UUS from a push start in the prone body position at different kick frequencies determined by a programmed metronome sounds	Two underwater sagittal view cameras with a frame rate of 100 Hz Six-level metronome sounds corresponding to the kick frequencies; 85, 90, 95, 105, 110 & 115%, generated by underwater speakers		
Shimojo et al. [48]	1 national swimmer (M)	24 yrs 176 cm 81.0 kg	41 × 15 s steady UUS in a swimming flume in prone body position (0.8 m s^−1^) (12–20 UUS cycles)	18 underwater cameras with a frame rate of 120 Hz used to obtain 3D coordinate data Two underwater cameras captured flow)	Microbubbles (50 μm) were used as tracer particles. A double-pulsed Nd:YAG laser was irradiated through the bottom of the flume to illuminate the flow area (wavelength 342 nm, maximum power P = 1 kW)	
Shimojo et al. [38] Experiment 1: Propelling efficiency assessment Experiment 2: Kinematic assessment	Experiment 1 17 national swimmers (9 M, 8F)	Male swimmers: 19.7 ± 1.1 yrs 176 ± 4 cm 70.9 ± 8.5 kg Female swimmers: 19.6 ± 0.8 yrs 161 ± 8 cm 55.7 ± 7.9 kg	2 × max UUS trials in prone body position (1 × with metronome device, 1 × with tape application aimed at restricting the swimmers ankle joints’ plantar flexors)	One underwater sagittal view camera with a frame rate of 60 Hz A tempo of 80% kick frequency was set in a waterproof metronome device		The ankle joint was taped to restrict plantar flexion. The active and passive plantar ankle flexions were measured on land
	Experiment 2 1 national swimmer (M)	20 yrs 171 cm 65.1 kg	2 × max UUS trials in prone body position (1 × with metronome device, 1 × with tape application aimed at restricting the swimmers ankle joints’ plantar flexors)	Six cameras around the swimmer with a frame rate of 120 Hz used to obtain 3D coordinate data A tempo of 80% kick frequency was set in a waterproof metronome device		
Wang and Liu [39]	10 international swimmers^a^ 10 regional club swimmers^a^	Elite: 22 ± 2 yrs 171 ± 6 cm 72 ± 6 kg Non-elite: 21 ± 1.8 yrs 171 ± 6 cm 65 ± 12 kg	3 × max UUS trials in prone body position	One sagittal view camera with a frame rate of 60 Hz		
Willems et al. [24]	26 national swimmers (15 M, 11F)	16.4 ± 2.5 yrs 174 ± 9.6 cm 61.7 ± 9.6 kg	3 × 10 m max UUS trials from a push start in prone body position. Feet were taped to restrict ankle movement	Four underwater cameras (sagittal, rear and bottom view) with a frame rate of 300 Hz 2D kinematic analysis		Goniometric measurements were used to determine ankle flexibility A hand held dynamometer measured ankle muscle strength
Yamakawa et al. [40]	8 national swimmers (F)	20.9 ± 1.9 yrs 163 ± 6 cm 54.9 ± 5.3 kg	15 m maximal UUS from a push start in the prone body position at different kick frequencies determined by a programmed metronome	Two underwater sagittal view cameras with a sampling frequency of 100 Hz Six-level metronome sounds corresponding to the kick frequencies; 85, 90, 95, 100, 105, 110 & 115%		
Yamakawa et al. [49]	8 national swimmers (M)	21.3 ± 0.7 yrs 173 ± 5 cm 70.3 ± 4.6 kg	3 × 25 m trials; undulatory swimming with a board, UUS and butterfly swimming. from a push start in prone body position 3 × trials; undulatory swimming with a board, UUS and butterfly swimming in a swimming flume at 80% velocity of 110%V. Participants executed 10 stroke cycles during each trial	Twenty above and underwater cameras 3D motion analysis		Active lower body range of motion

^a^participant sex was not specified, yrs years, cm centimetres, kg kilogram, Hz hertz, 3D three-dimensional, M male, F female, max maximal, UUS undulatory underwater kick

Correlation and regression analyses were one of the most commonly performed statistical approaches used in the eligible studies (Table 4). Across the nine studies that performed correlation analyses, UUS velocity was found to be significantly correlated with at least one kinematic outcome in eight [6, 7, 10, 15, 34, 36, 37, 41] and at least one kinanthropometric outcome in four studies [6, 10, 24, 41]. The most common significantly correlated kinematic variables to UUS velocity were kick frequency [6, 7, 37], vertical toe velocity [10, 15], knee angular velocity [15, 34] kick amplitude [36, 37] and angle of attack for the trunk, thigh and foot [36, 37]. While the strength of these correlations was typically strong to nearly perfect in magnitude, there were between-study variation for kick frequency (*r* = 0.43–0.90). Two of these studies also performed regression analyses to obtain greater insight into determinants of UUS velocity [34, 36].

**Table 4:** Summary of study kinematic and kinanthropometric correlations to underwater kick velocity

Reference	Kinematic variables (a)	Kinanthropometric variables (b)	Correlation to UUS velocity		Regression (*r*^2^)
			a	b	
Alves et al. [6]	Foot resultant acceleration Kick frequency	Transverse elbow amplitude	0.94** 0.90*	0.90*	
Atkison et al. [10]	Max vertical toe velocity(UK) Horizontal kick displacement(DK) Max vertical toe velocity(DK:UK)	Max knee flex/ext angle Max knee ext angle Max ankle flex/ext angle Max chest flex angle Max chest flex/ext angle Max ankle ext angle	0.63* 0.63* − 0.73*	0.88* 0.84* 0.67* 0.61* 0.52* 0.45*	
Connaboy et al. [34]	Max knee angular velocity Max knee angular velocity, max ankle angular velocity and knee ROM Max knee angular velocity, max ankle angular velocity and knee ROM		0.63***		0.94 (participant as fixed factor) 0.40 (no fixed factor)
Higgs et al. [15]	Peak vertical toe velocity Body wave velocity Peak hip angular velocity (UK) Mean knee angular velocity (UK) UK duration		0.85* 0.78* 0.73* − 0.63* − 0.79*		
Hochstein and Blickhan [7]	Kick frequency		0.43**		
Houel et al. [36]	Hip: Angle of attack (thigh) + Phase time (ankle) COM: Angle of attack (thigh) + Phase time (ankle) COM: Angle of attack (foot) COM: Phase time (Knee) Hip: Kick frequency Hip: Angle of attack (trunk) COM: Phase time (ankle) COM: Kick Amplitude				0.89*** 0.79** 0.7** 0.68** 0.68** 0.56** 0.52* 0.43*
Houel and Elipot [37]	Kick frequency (7 m) Angle of attack (leg) (5.5 m) Angle of attack (thigh) (6.5 m) Angle of attack (trunk) (5.5 m) Angle of attack (foot) (6 m) Kick amplitude (5.5 m)		0.67* − 0.63* − 0.65* − 0.65* − 0.65* − 0.66*		
Ikeda et al. [41]	Shoulder (°) Lower trunk (°) angular displacement in deceleration phase Upper leg (rad/s) angular displacement in acceleration phase Ankle (m) Lower leg (°) Lower trunk (°) angular displacement in acceleration phase Inferior end of the rib (m) Relative coordinate value to GT shoulder (m) Lower trunk (°)		0.80* 0.68* − 0.67* − 0.68* − 0.70* − 0.72* − 0.87** − 0.87** − 0.91**		
Willems et al. [24]		Dorsi flex strength Ankle internal rotation strength		0.53* 0.47*	

Values for each study are listed from highest to lowest correlation

Kinematic variables (a), Kinanthropometric variables (b), UUS undulatory underwater kick, UP up kick, DK down kick, max maximal, flex flexion, ext extension, ROM range of motion

*p ≤ 0.05, **p ≤ 0.01, ***p < 0.001

Two of the three studies reporting kinanthropometric results utilised active range of motion measures during the UUS [6, 10], whereas another study assessed single joint muscular strength measures [24]. The strength of the correlations for the active range of motion measures (*r* = 0.45–0.90) was larger than those for the muscular strength measures (*r* = 0.47–0.53), strong to very large, and moderate to strong, respectively.

Table 5 provides a summary of some of the primary biomechanical descriptors of the UUS phase, with an emphasis on how these may change as a function of body position, horizontal distance from the starting block and across different levels of swimmers. The majority of studies reported UUS velocity [6, 7, 16, 17, 24, 33–37, 39–41, 48, 49], kick frequency [6, 7, 16, 24, 33–38, 40, 41, 49] and kick amplitude [6, 7, 15, 24, 34–37, 40, 41, 48, 49].

**Table 5:** Summary of study underwater kick performance variables data most frequently correlated with velocity

References	Participants	UUS Velocity (m s^−1^)	Kick Frequency (Hz)	Kick Amplitude (m)	Vertical Toe Velocity (m s^−1^)	Knee Angular Velocity (° s^−1^)	Knee Range of Motion (°)
*Body position*							
Alves et al. [6]	6 junior national swimmers	Prone: 1.46 ± 0.15 Dorsal: 1.42 ± 0.21 Lateral: 1.27 ± 0.11	Prone: 2.35 ± 0.27 Dorsal: 2.30 ± 0.33 Lateral: 2.08 ± 0.36	Prone: 0.50 ± 0.06 Dorsal: 0.55 ± 0.08 Lateral: 0.59 ± 0.09			Prone Knee Flex: 119.34 ± 3.70 Dorsal Knee Flex: 120.72 ± 13.05 Lateral Knee Flex: 107.73 ± 8.68
Arellano et al. [33]1999	11 national swimmers (M)	Prone: 1.69 Dorsal: 1.67	2.22 2.25				Prone: DK: 169.18 UK: 171.00 Dorsal: DK: 118.27 UK: 114.27
*Horizontal distance from starting block*							
de Jesus et al. [35]	4 international swimmers	BSFI 1st 4 kick cycles: 1.47 ± 0.11 Last 4 kick cycles: 1.28 ± 0.07	2.42 ± 0.15 2.33 ± 0.19	0.61 ± 0.07 0.55 ± 0.05			
		BSFE 1st 4 kick cycles: 1.44 ± 0.04 Last 4 kick cycles: 1.30 ± 0.04	2.41 ± 0.20 2.39 ± 0.24	0.60 ± 0.06 0.55 ± 0.07			
Houel et al. [36]	12 national swimmers	At 5.5 m: 2.18 ± 0.21 At 7.5 m: 1.76 ± 0.15	At 7.5 m: 2.32 ± 0.21	At 7.5 m: 0.71 ± 0.60			
Houel and Elipot [37]	10 national swimmers	At 6 m: 1.99 ± 0.13 At 6.5 m: 1.93 ± 0.14 At 7 m: 1.74 ± 0.25 At 7.5 m: 1.76 ± 0.17	At 7.5 m: 2.32 ± 0.22	At 7.5 m: 0.71 ± 0.60			
*Level of swimmer*							
Connaboy et al. [34]	17 national swimmers (8 M, 9F)	1.20 ± 0.13	2.13 ± 0.23	Hip: 0.13 ± 0.03 Knee: 0.27 ± 0.04 Ankle: 0.46 ± 0.06 5th MPJ: 0.61 ± 0.07		702.7 ± 82.9	89.6 ± 6.9
Higgs et al. [15]	7 national swimmers (7 M, 3F)			DK: 0.58 ± 0.09 UK: 0.58 ± 0.09	− 3.61 ± 0.63 4.10 ± 0.63	260.0 ± 28.9 − 190.1 ± 43.6	
Hochstein and Blickhan [7]	2 national swimmers (F)	Subject 1: 1.22 ± 0.06 Subject 2: 1.18 ± 0.06	1.98 ± 0.10 2.13 ± 0.10	0.54 ± 0.04 0.52 ± 0.03			
Hochstein and Blickhan [17]	4 national swimmers (F) 6 regional club swimmers (3 M, 3F)	1.23 ± 0.04 1.09 ± 0.13		Toe: 0.22 ± 0.01 Toe: 0.24 ± 0.06			
Ikeda et al. [41]	9 swimmers (M)	1.75 ± 0.16	2.37 ± 0.23				109.0 ± 10.8 (min)
Shimojo et al. [16]	10 national swimmers (M)	1.60 ± 0.12	2.26 ± 0.16				
Shimojo et al. [16]	1 national swimmer (M)		1.14 ± SD to 1.30 ± SD across 41 trials	0.70 ± 0.04 to 0.74 ± 0.02 across 41 trials			
Shimojo et al. [48]	Experiment 1 17 national swimmers (9 M, 8F)	1.33 ± 0.19	1.65 ± 0.18	0.57 ± 0.06			
	Experiment 2 1 national swimmer (M)						
Wang and Liu [39]	10 international swimmers 10 regional club swimmers	3.34 ± 0.51 2.10 ± 1.22				Significantly greater in international team than regional club level (no numerical data provided)	
Willems et al. [24]	26 national swimmers (15 M, 11F)	1.64 ± 0.20	2.08 ± 0.40				
Yamakawa et al. [40]	8 national swimmers (F)	1.35 ± 0.08	1.99 ± 0.15	0.48 ± 0.05	DK: − 1.91 ± 0.14 UK: 1.68 ± 0.20		
Yamakawa et al. [49]	8 national swimmers (M)	1.19 ± 0.09	1.59 ± 0.23	0.31 ± 0.07		DK: 390.8 ± 59.8 UK: − 504.0 ± 67.9	73.3 ± 6.6

*UUS* undulatory underwater kick, *UK* up kick, *DK* down kick, *M* male, *F* female, *BSFE* backstroke start with feet emerged, *BSFI* backstroke start with feet immerged

Alves et al. [6], and Arellano et al. [33] collected data in prone, dorsal and ventral body positions and compared UUS velocity, kick frequency, kick amplitude and knee range of motion across the different body positions. The UUS velocity (prone: 1.46 and 1.69 m s^−1^; dorsal: 1.42 and 1.67 m s^−1^) and frequency (prone: 2.35 and 2.22 Hz; dorsal: 2.30 and 2.25 Hz) were similar in both the prone and dorsal body positions, with both positions substantially greater than in the lateral position. Alves et al. [6] reported values for kick amplitude for each body position (prone: 0.50 m; dorsal: 0.55 m).

de Jesus et al. [35], Houel, Elipot, Andree and Hellard [36], and Houel et al. [37] sought to examine how UUS velocity, kick frequency and amplitude may change as a function of horizontal distance from the starting block. In backstroke starts, UUS velocity decreased from the first four kick cycles to the last four cycles prior to resurfacing at 15 m [35]. The reductions in UUS velocity appear to reflect declines in both kick amplitude and kick frequency that were observed from the first four to the last four kick cycles [35]. Houel and colleagues [36, 37] also saw a consistent decrease in velocity as the swimmer approached the 15 m mark.

Several studies also reported data for different levels of swimmers. As expected, the UUS velocity was typically greatest for international swimmers, followed by national and regional swimmers [17, 38, 39]. Higher UUS velocities were generally associated with higher kick frequencies and consistent kick amplitudes [7, 17, 24, 34, 38, 40]. Two studies examined other kinematic factors that may be related to UUS velocity, kick frequency or kick amplitude. Wang and Lui [39] reported that the international swimmers had a significantly greater UUS knee angular velocity than the regional-level swimmers; however, no numerical data were provided for either group of swimmers. Yamakawa et al. [49] also provided data for knee angular velocity during the up and down kick phases, as well as knee range of motion during the UUS in national-level male swimmers (Table 5).

The study conducted by Shimojo et al. [47] had 15 national swimmers (10 M, 5F) who perform two tasks, one that required 10 × 10 m UUS trials at different kick frequencies determined by a target sound, with the second task being identical with the exception of having no sound. The results, that were reported as timing error (s) and displacement error (%), indicated that providing a target sound as a form of auditory augmented feedback improved their timing (which may improve their ability to maintain an optimal kick frequency), but that this feedback had less influence on their displacement measures [47].

Four studies reported data in relation to the net, drag and/or reaction forces, as well as the coefficient of drag associated with aspects of the UUS (Table 6) [42, 43, 45]. Lyttle et al. [43] examined how the net and drag forces would change at different velocities when the swimmer was passively towed underwater at a depth of 0.6 m. As the velocity increased from 1.6 m s^−1^ up to 3.1 m s^−1^, the net force became increasingly negative as a result of the drag force increase in magnitude. While Jensen and McIIwain [45] reported a drag force and reaction force, the description of their methods was poor, with no other relevant data provided. Hochstein et al. [44] reported the coefficient of drag during the glide and undulatory phases of UUS and compared the respective results between computational fluid dynamics (CFD) and experimental conditions. The study saw flow field similarities; however, the experimental results for the undulatory phases were not specified. CFD showed a much larger coefficient of drag during undulatory underwater swimming compared to gliding [44].

**Table 6:** Summary of study kinetic data

References	Participants	Trials and methodology	Velocity (m s^−1^)	Net force (N)	Drag force (N)	Drag coefficient (*C*_D_)		Reaction force (*N*)
Hochstein et al. [44]	1 national swimmer (F)	20 m max UUS trial from a standing start in the prone body position and 2D Particle Image Velocimetry (PIV) Drag coefficient was compared between computational fluid dynamics (CFD) and experimental conditions, and the analysis was divided into **glide** and **undulatory** phases				CFD	Exp	
						0.3	0.25–0.29	
						2.98	NA	
Jensen and McIlain [45]	2 international swimmers (1 M, 1F)	No information was provided on the number of trials, distance of each trial, trial goals, e.g. max speed or percentage of normal kick frequency performed. Anthropometric measurements were taken of the lower extremity of the swimmers and used to formulate a geometric representation of the segments’ inertial properties. Drag forces, joint forces and moments of force were then calculated using the segment inertial properties and kinematic data of the UUS			− 45.5			92.35
Lyttle and Blanksby [42]	Study 1: 40 national swimmers (M) Study 2: 16 national swimmers (M)	Study 1: Towed in a prone position 25 m at a depth of 0.6 m underwater. At this depth, swimmers were towed at 1.6; 1.9; 2.2; 2.5 and 3.1 m s^−1^. **Drag force** was recorded using a unidirectional load cell Study 2: Towed in a prone position 25 m at a depth of 0.5 m underwater at velocities 1.6; 1.9; 2.2; 2.5 and 3.1 m s^−1^. **Net force** was recorded using a unidirectional load cell	1.6	21.3 ± 12.6	58.1 ± 9.3			
			1.9	− 48.3 ± 14.8	80.4 ± 10.0			
			2.2	− 87.0 ± 18.3	109.4 ± 11.1			
			2.5	− 122.1 ± 20.0	140.5 ± 14.4			
			3.1	− 192.7 ± 22.0	204.1 ± 19.2			
Lyttle et al. [43]	16 national swimmers	Towed in a prone position 25 m underwater at velocities 1.6, 1.9, 2.2, 2.5 and 3.1 m s^−1^ performing prone undulatory kicking. During each trial, **net force** was recorded using a unidirectional load cell	1.6	− 21.3 ± 12.6				
			1.9	− 48.3 ± 14.8				
			2.2	− 87.0 ± 18.3				
			2.5	− 122 ± 20.0				
			3.1	− 193 ± 22.0				

UUS undulatory underwater kick

*p ≤ 0.05

Five of the 25 studies included in this systematic review reported results related to UUS hydrodynamic mechanisms, including vortices, jet flow and wake [10, 14, 38, 44, 46] (see Table 7). Two of these studies used a swimming flume [38, 46], whereas the other two studies obtained 2D kinematic data from 3 × 15 m trials to calculate the hydrodynamic outcomes [10, 14]. Hochstein et al. [44] compared hydrodynamic mechanisms between CFD and experimental conditions during the glide and undulatory phases.

**Table 7:** Summary of study hydrodynamics data

Reference	Trials	Hydrodynamic mechanism
Atkison et al. [10]	3 × 15 m max UUS from a push start in prone body position 2D kinematic analysis	Peaks in horizontal velocity occurred at the same time as, or immediately following peaks in vertical toe velocity. Furthermore, there was a greater increase in horizontal velocity for the down kick (1.67 m s^−1^, *r* = 0.983*) than the up kick (1.62 m s^−1^, *r* = 0.993*), corresponding to faster peak vertical toe velocities during the down kick phase (DK = − 2.38 m s^−1^, UK = 1.99 m s^−1^). The authors interpreted these findings to suggest an association between magnitude of peak vertical toe velocity and vortex magnitude, and timing of peak vertical toe velocity and timing of vortex shedding, based on the idea that efficient swimmers create a large static vortex at the end of the down kick and a small vortex at the end of the up kick
Elipot et al. [14]	3 × 15 m max UUS from a grab start in prone body position 2D kinematic analysis	By increasing kick amplitude, swimmers create a bigger wake of counter-rotation vortices that contribute to the leg propulsive forces. However, when kick amplitude is increased, the swimmer’s form drag will also increase
Hochstein et al. [44]	20 m max UUS trial from a standing start in the prone body position 2D Particle Image Velocimetry (PIV)	Resulting vortex rings after the up and down kick merge into longitudinal vortex strings in the swimmer’s wake Increased vortex generation indicates increased drag
Miwa et al. [46]	5 × steady UUS in a swimming flume (1.0 m s^−1^) 2D flow analysis	The results confirm the existence of a pair of vortices and jet flow in the wake of undulatory kicking motion. After the upward motion, some pairs of small vortices and the jet flow were also confirmed; however most were from the down kick The swimmer created the vortex ring for propulsion
Shimojo et al. [48]	41 × 15 s steady UUS in a swimming flume in prone body position (0.8 m s^−1^) (12–20 UWK cycles)	During the downward kick, the lower limbs moved downwards with internal rotations and ankle plantar flexion, and the pressure difference between the dorsal and ventral side produced a fluid force The pressure difference produced a leading edge vortex that travelled from the ventral to dorsal side of the feet through the toes. After a clockwise rotating vortex generated by the leading edge, the vortex was shed from the foot, inducing downstream flow. The shedding of vortices from the feet expanded and created a cluster The swimmer externally rotated his lower limbs at the end of the downward kick, and the toes of the feet approached and then separated each other. This action generated a strong cluster of vortices and jet flow in the wake resulting in thrust The cluster of shed vortices and jet flow were released from the feet after the downward kick, and moved towards to the ventral side of the swimmer During the upward kick, upstream flow was created with small vortex structure

*UUS* undulatory underwater kick, *2D* two dimensional, *DK* down kick, *UK* up kick

*p ≤ 0.05 can be considered as significant

## Discussion and Implications

The purpose of this systematic review was to critically appraise the current peer-reviewed literature on how biomechanical factors might influence UUS performance. The primary finding of the current systematic review was that a range of variables may be strong correlates or predictors of UUS velocity. A number of these UUS variables were also found to vary as a function of body position (prone and dorsal), horizontal distance from the starting block and level of swimmer, with such differences potentially explained as a result of variations in the vortices in the wake surrounding the swimmer. While this review provides a systematic analysis of our current understanding of the kinematics, kinetics and hydrodynamics impacting UUS, no long-term training intervention research exists to provide an understanding of how chronic changes in biomechanical and/or kinanthropometric factors may interact with each other and contribute to alterations in UUS velocity and overall swim performance.

### Performance Determinants Correlated with UUS Velocity

As a cyclical human activity, it could be hypothesised that a kick frequency would result in greater UUS velocity. Consistent with this hypothesis, the results of this review indicated that a range of UUS kinematic and kinanthropometric outputs that either directly or indirectly correspond to kick amplitude and kick frequency characteristics had large (*r* = 0.5–0.7), very large (*r* = 0.7–0.9) or nearly perfect (*r* > 0.9) correlations to UUS velocity. The strongest correlations to UUS velocity were foot resultant acceleration (*r* = 0.94) and kick frequency (*r* = 0.90), reported by Alves et al. [6] in a group of six junior national swimmers. Very large correlations to UUS velocity were also found between several kinematic variables, peak vertical toe velocity (*r* = 0.85), shoulder angle (*r* = 0.80), body wave velocity (*r* = 0.78) and peak hip angular velocity (*r* = 0.73), and measures of the maximum knee flexion and extension angle (*r* = 0.88) and maximum knee extension angle (*r* = 0.84) [10, 15, 41].

However, the study conducted by Ikeda et al. [41] reports that there was no significant correlation between kick frequency and velocity (*r* = 0.28); however, a significant relationship between kick frequency and the duration of the deceleration phase (*r* = − 0.842) was observed. Such results suggested that the more time a swimmer spends in the up kick phase, the lower the kick frequency, which may be indicative of utilising a higher kick amplitude to drive thrust and propulsion. Evidently, the time of the deceleration phase correlated with the relative vertical velocity of the shoulder to greater trochanter at maximal swimming velocity.

#### Vertical Toe Velocity and UUS

Peak vertical toe velocity during the up kick and down kick can explain approximately 72.3% of the variance in UUS velocity (*r* = 0.85) [15]. Peaks in horizontal UUS velocity have been found to occur at the same time, or immediately after peaks in vertical toe velocity, and this increase in forward speed is observed to be more apparent during the down kick, illustrated by a strong correlation between foot resultant acceleration and UUS velocity (*r* = 0.94) [6, 10, 49]. The similar timing of the peak in vertical toe velocity and UUS velocity may reflect a strong relationship between the magnitude and timing of maximum vertical toe velocity, and that of horizontal acceleration [23, 33, 46, 50, 51].

Atkison et al. [10] reported in a study of regional and international male swimmers that average vertical toe velocity during the up kick showed the highest correlation to the swimmer’s centre of mass velocity (*r* = 0.63), suggesting that faster swimmers are more proficient at the up kick than slower swimmers [10]. Additionally, significant correlations were identified between up kick velocity and horizontal velocity (*r* = 0.99) as well as down kick velocity and horizontal velocity (*r* = 0.98) [10]. Higgs and colleagues [15] also found that vertical toe velocity (*r* = 0.85) and up kick duration (− 0.79), supported by results of Ikeda et al. [41] were significantly correlated with UUS velocity, as well as body wave velocity, peak hip extension angular velocity and mean knee flexion angular velocity [15, 41].

#### Angular Velocities and UUS

The up kick is characterised by hip extension and knee flexion [10], with better swimmers typically extending at the hip before initiating knee flexion [50] suggesting that a high hip angular velocity may have a positive influence on thrust production and performance [15]. Mean hip (DK: − 85.0 ± 33.2 deg s^−1^; UK: 79.0 ± 32.5 deg s^−1^) and peak hip (DK: − 191.9 ± 61.4 deg s^−1^; UK: 248.0 ± 44.8 deg s^−1^) angular velocity and mean knee (DK: 260.0 ± 28.9 deg s^−1^; UK: − 190.1 ± 43.6 deg s^−1^) and peak knee (DK: 532.8 ± 36.9 deg s^−1^; UK: − 237.5 ± 40.5 deg s^−1^) angular velocity values suggest that the knee action contributes more than the hip action during the down kick, whereas the contribution of the hip and knee is similar during the up kick [15]. These findings align with that of Arellano et al. [50] and Atkison et al. [10], where elite swimmers utilise hip extension in the up kick to a greater extent than novice performers, highlighting the importance of hip extension. Further, Ikeda et al. [41] identified in their study of nine elite male swimmers that faster swimmers moved their lower trunk with greater angular displacement during the acceleration phase (down kick) than the slower swimmers (fast swimmers: 25.2°, slow swimmers: 4.5°), where the angular displacement of the lower trunk was also correlated with the angular displacements of the shoulder, knee, upper leg and lower leg. Despite the importance of the lower trunk angular displacement, excessive lower trunk movement may increase frontal projection area which in turn creates greater water resistance [14, 41].

Connaboy et al. [34] found in their parsimonious model for maximal swimming velocity that maximum knee angular velocity, ankle angular velocity and knee range of motion accounted for a very large amount of variance in UUS velocity (adjusted *r*^2^ = 0.93). After removing the participant as the fixed factor from the analysis, the strength of the prediction was reduced (adjusted *r*^2^ = 0.40). As a result, maximum ankle angular velocity and knee range of motion were no longer statistically significant, leaving only maximum knee angular velocity as a significant predictor of UUS velocity. The results of these two regression analyses support the importance of maximum knee angular velocity during UUS, but also that an individual’s kinanthropometry and/or technique may influence the strength of the relationship between joint angular velocity and range of motion to that of their UUS velocity [34]. For example, a high mean knee angular velocity allows the swimmer to complete the latter phase of the up kick phase quickly to minimise their hydrodynamic resistance [15, 34] and also increase kick frequency by reducing the duration of each kick cycle. Subject-specific analysis [52] should be employed to consider the importance of individual UUS techniques when interpreting data [34].

#### Body Wave Velocity and UUS

A stepwise regression analysis revealed that maximum vertical toe velocity and body wave velocity were strong predictors of UUS velocity (*r*^2^ = 0.72), with an additional 5.2% explained by the mean body wave velocity (partial correlation *r* = 0.46) [15]. Undulatory locomotion is accomplished via oscillations that pass along the length of a swimming body, and an understanding of the temporal sequencing between these oscillations as they occur is fundamental to appreciating how UUS velocity can be optimised. The oscillations generate a wave that transfers momentum to the surrounding fluid resulting in propulsive impulse and thrust [15, 20]. The composition of the body oscillations and their relationships determine the shape and velocity of the propulsive waveform, subsequently impacting UUS forward motion [25, 53–55], where shape refers to the frequency, amplitude and temporal coupling of the undulatory movements [16, 54]. Research conducted by Ikeda et al. [41] reported that the movement of the shoulder, lower trunk and lower leg along the body wave was associated with maximum horizontal UUS velocity. As body wave velocity and vertical toe velocity both have a level of independence and are individually related to UUS velocity, optimal UUS performance will only be achieved if the athlete is able to produce high body wave and vertical toe velocity [15].

### Hydrodynamics and UUS Parameters

Vortices are integral to the production of propulsive force and minimisation of drag during the UUS [33, 50, 53]. Vortices are described as rotating masses of water that are created by the heaving and pitching motions (cyclic motion) of the toes during UUS [25, 50]. Vortices represent the transfer of momentum from the water to the body, and vice versa, resulting in body acceleration [10, 56]. Higher vertical toe and body wave velocity and vertical toe velocity have been linked to vortex creation and thrust production, with these vortices having the potential to explain some of the differences in athlete’s UUS velocity [10, 15, 56]. The flow pattern behind the swimmer in the wake shows a jet stream between two counter-rotational vortices following the down kick [7, 44]. Similar results were found by Hochstein et al. [44], Miwa et al. [46] and Shimojo et al. [38], who confirmed the existence of a pair of vortices and jet flow in the wake of the swimmer, which are assumed to be a part of a vortex ring or structure that drives propulsion. The majority of propulsive force during the UUS was shown to be generated at the end of the down kick, where swimmers externally rotated their lower limbs and moved their feet closer together [38, 57]. This action subsequently generates a strong cluster of vortices and jet flow, increasing the water momentum in the wake, resulting in thrust [38, 44].

### Kinanthropometry and UUS Parameters

Kick amplitude reflects the actions of the hip, knee and ankle, where a larger range of motion at these joints creates bigger counter-rotation vortices that maximise leg propulsion to drive forward motion [14]. The results of the current systematic review reflect a positive relationship between maximum knee (*r* = 0.88), ankle (*r* = 0.67) and chest (*r* = 0.52) flexion and extension angles and UUS velocity, with results for the hip not reported [10]. Optimisation simulations of UUS predict that swimmers who exhibited greater similarity between maximum joint range of motion, specifically extension and flexion angles would have higher average horizontal velocity [58]. This was evidenced in the study conducted by Ikeda et al. [41], where a greater shoulder angle was significantly correlated with UUS velocity (*r* = 0.80), as this improves streamline position and decreases effects of drag. Similarly, Atkison et al. [10] observed the importance of high levels of upper thoracic flexibility for UUS velocity, as this flexibility is required to dampen body undulations of the lower segments and reduce resistive drag by maintaining a small angle of attack of the arms. Wang [39] suggests that the angular velocity of the trunk may also contribute to the higher kick frequency seen in skilled swimmers, describing how the undulatory wave that is initially observed at the lower trunk may enhance peak angular velocities of the lower limb joints. Ikeda et al. [41] also observed that faster swimmers moved their lower trunk with greater angular displacement, which further increased the angular displacement of the shoulder, knee and lower leg. The results suggest that greater angular displacement of the lower legs and feet may produce thrust and in turn propulsion [41]. However, if such movements become too large, this may lead to greater water resistance due to greater frontal projected area [41].

UUS velocity might be achieved in multiple ways, at one extreme, through large undulatory movements (higher amplitude) to maximise propulsive impulse production with high active drag (high energy requirement/cost) or small movements (smaller amplitude) that produce a reduced amount of propulsive impulse but minimise drag [34]. Hochstein and Blickhan [17] reported that slower swimmers utilised higher kick amplitudes and initiated the undulatory wave at the hands. In the slower swimmers, the larger mass of water being propelled by a higher kick amplitude was negated by a lower kick frequency and increasing drag, which resulted in reduced overall velocity. Consistent with this view, Elipot et al. [14] have found that higher-level swimmers utilise strong joint synergy between the hip, knee and ankle joint, whereby they adopt a regulation loop in which the hip-ankle and the knee action are independent and control the kick amplitude. Skilled athletes appear able to better maximise propulsive impulse during the UUS by employing optimal amplitudes of the end effector that in turn minimise flow separation and maximise energy reuse from the vortices in the wake around the body [59–61]. How variations in a swimmer’s kinanthropometric and technical characteristics may influence the combination of joint movements that optimise the relationship between kick amplitude and kick frequency and their resulting UUS velocity remains unclear.

While the coordination of multiple body joints is important to optimise the undulatory movements that maximise UUS velocity, the swimmers’ flexibility across these joints has the potential to influence the swimmers’ coordination patterns, efficiency of thrust production and ability to minimise resistance forces [24]. However, the optimal ranges of motion across multiple joints for maximising UUS velocity still remain unclear [24, 62–65].

Few studies have investigated the relationship between ankle flexibility and UUS performance, with the results being somewhat equivocal [24, 48]. When ankle plantarflexion range was restricted by tape, it had a negative impact on kick frequency, movement efficiency and UUS velocity [24]. Similarly, Shimojo et al. [48] found that when restricting ankle plantarflexion range, there was a significant decrease in velocity due to the inhibition of the rotational function; however, Froude efficiency (a measure of swimming efficiency) remained unchanged. The hydrodynamic force acting on the foot during active range of motion was thought to be higher than expected as the maximal plantarflexion angle did not decrease following tape application [48]. Shimojo et al. [48] concluded that foot rotational ability, rather than ankle flexibility is associated with increased UUS velocity, appearing consistent with the results of Willems et al. [24].

There was no significant correlation between dryland ankle flexibility measures and UUS velocity [24]. Alternatively, Willems et al. [24] observed positive correlations between dorsiflexion strength (*r* = 0.53) and ankle internal rotation strength (*r* = 0.47) to UUS velocity. While the ankle internal rotation strength correlation was expected, the importance of dorsiflexion was unexpected as ankle plantar flexion produces propulsive force to drive thrust and momentum [10]. However, dorsiflexion strength may also play a role during the down kick to maintain a rigid foot position while applying force to the water [10]. Yamakawa et al. [49] suggested that the internal/external rotations of the hip joint may contribute to the control of the direction of the dorsal side of the ankle and foot, therefore identifying this as an area of interest in understanding the UUS.

### Optimising Kick Frequency and Amplitude

A number of studies have looked to examine how acute changes in kick frequency may influence UUS velocity [16, 34]. To increase their kick frequency beyond their preferred frequency, swimmers must generate larger torque power and/or reduce resistance forces [16]. As increasing joint torques would require more internal work [66], swimmers may not be able to maintain their preferred kick amplitude when increasing their kick frequency for long periods, resulting in either maintenance or reduction in UUS velocity [16]. Such results appear consistent with the literature that higher swim velocities are associated with increased kick frequency and a maintenance of kick amplitude [16, 19, 36, 37, 67]. For undulatory swimming with preferred kick frequency, the amplitude in the end effector is about one fifth of the body length [21]. Hochstein et al. [21] reported that this amplitude range of 0.2 – 0.3 body lengths can be considered a fixed (physical) constraint, regardless of body size, shape or movement.

In their regression analyses, Connaboy et al. [34] observed that the reduction in explained variance with the removal of participant as the fixed factor may be indicative of the number of possible solutions to the task (maximise UUS velocity) in relation to the individual’s own organism constraints [68]. These emphasise that an athlete’s optimal movement combination when performing UUS may be somewhat different to others, as each swimmer needs to exploit their own organism constraints to maximise propulsive impulse while simultaneously reducing drag in response to the task and environmental constraints [68]. For example, Connaboy et al. [34] reported that in a sample of 17 national swimmers (8 M, 9F), two swimmers had identical mean maximal swimming velocity (1.18 m.s^−1^). Swimmer A had the lowest kick amplitude of the entire group (0.52 m), but the second highest kick frequency (2.22 Hz). In contrast, Swimmer B had the second highest kick amplitude (0.69 m) but third lowest kick frequency (1.98 Hz). These results provide some preliminary data that swimmers who possess different organism-level constraints, e.g. force production or range of motion capabilities at specific joints, or limb and body length may utilise different movement solutions when performing the UUS [34]. Future research should therefore examine how a combination of factors such as a swimmer's kinanthropometry and technique as well as the swimming stroke and race distance may also alter the movement solutions generated to optimise UUS velocity [21, 34].

### Influence of Body Position on UUS Performance Variables

The UUS is a skill utilised in all swimming strokes, with the exclusion of breaststroke. The prone body position is utilised in the front crawl and butterfly, with the dorsal body position seen in backstroke. Prone UUS velocity was highly correlated with kick frequency (*r* = 0.90) [6] and down kick acceleration decreased significantly during a dorsal body position. During prone and dorsal UUS, a qualitative analysis of the kicking path showed near identical trajectories (amplitude and timing), as well as up and down kick duration [33]. The two studies comparing UUS performance in the dorsal prone position reported minor differences in the UUS velocity, kick amplitude and frequency in six junior national swimmers (17.02 ± 0.36 years) [6] and 11 male national swimmers (19.9 ± 2.15 years) [33]. Similar results were also obtained for kick length and mean body speed between prone and dorsal UUS [33]. Towards the end of knee flexion in both body conditions (the up kick for prone UUS and down kick for dorsal UUS), there were significant differences in the shoulder (P: 163.90°, D: 159.36°, p < 0.05) and knee angle (P: 118.27°, D: 114.27°, p < 0.05), where there was a larger shoulder and knee angle during the prone position [33]. Dorsal kicking seemed to require greater levels of plantarflexion [6], and Arellano et al. [33] described UUS in this body position to have more pronounced body oscillations than in the prone position.

When the swimmer adopts a non-prone UUS position (i.e. dorsal or lateral), body undulations are more evident, which may imply a difference in kicking technique, propulsive force production, joint range of motion and/or resistance forces encountered. This could be related to the prone body position providing a more stable position for the swimmer’s centre of mass compared to the dorsal body position, which may contribute to a more efficient movement pattern [6]. Theoretically, the prone position may allow greater UUS velocity to be generated as a result of its higher kick amplitudes and frequencies and/or reduced resistance forces than other swimming positions. While such results have been shown when comparing prone and dorsal positions to a lateral position, only relatively minor differences have been discovered between prone and dorsal positions [6, 33].

### The Impact of Horizontal Distance and Depth on UUS Variables

When entering the water after a dive start, the swimmer’s velocity (~ 3.61 m.s^−1^) is greater than at any other time of the race due to the resistance of the water being greater than that of air [35, 69]. de Jesus et al. [35] reported that UUS velocities decreased from the first four kick cycles to the final four before surfacing, with this associated with a reduced kick amplitude and frequency. Determining when and where the underwater swimmer should begin kicking compared to maintaining a streamlined glide position is an important practical question [35].

Lyttle and Blanksby [42] suggest that swimmers should perform the glide at approximately 0.4 m underwater at all velocities above 1.9 ms^−1^ to gain maximum drag reduction benefits, where a 15–18% reduction in total drag was found when compared to swimming at the surface. It has been proposed that propulsive movements should be initiated when the underwater velocity drops to 1.9–2.2 m.s^−1^ because this is the maximum range of speeds that produced a significant reduction in net force in the kicking conditions compared to streamlined positions [43]. Using this recommendation, Elipot et al. [14] and Houel and colleagues [37] suggest that high-performance swimmers should initiate undulatory movements, using the legs to generate propulsion when the centre of mass is ~ 6 m from the starting wall. At this distance, mean velocities were 1.99 ± 0.13 m.s^−1^, aligning with findings of Lyttle et al., [43] and Elipot et al. [14]. Houel and Elipot [37] conclude from their results of a multiple stepwise regression and comparison to results of other studies when a swimmer initiates the UUS after completing the glide phase, he/she can maximise their UUS velocity by progressively increasing kick frequency and maintaining their kick amplitude [35].

### Methodological Considerations

As with any systematic review or meta-analysis, there are a number of strengths and limitations that may affect the generalisability of the results. These strengths and limitations are summarised with respect to the review methodology as well as the reviewed literature.

#### Review Methodology

The primary strengths of the review were the pre-registration of the review methodology, the use of the PRISMA methodology and use of a risk of bias tool recently developed for cross-sectional biomechanical studies [1]. The primary limitation of the review process was the restriction of articles to those written in English and that were peer-reviewed journal articles or longer conference proceedings articles. Only articles about human swimmers were included which may limit our understanding of the UUS as other studies involving animals or computational simulation approaches may also have provided some insight into improving UUS velocity in human swimmers.

#### Measurement of UUS

In terms of the literature, the major strength of this review compared to that of the previous review by Connaboy et al. [34] is the identification of an additional 17 studies. The 23 studies included in the current systematic review exhibited some degree of inconsistency with the measurement of UUS kinematic measures, specifically the distances of the underwater phase (10 m, 15 m, 20 m or 25 m) and starting positions of each trial. The majority of studies [10, 16, 17, 24, 33, 35, 36, 40, 47] used a push start to 10 m or 15 m; however, some studies analysed the UUS following a block start [6, 14, 36]. As a result, determining the correlation of biomechanical parameters to underwater velocity may be influenced by the studies' measurement distance and their approach to determining UUS performance.

There also appear to be some differences in the nature of the swim task performed across the included studies. Within this review, the majority of the studies tested the swimmers in a competition swimming pool and had them perform UUS to a set distance [6, 7, 10, 14–17, 24, 33–37, 40, 41, 47, 48]. Other studies looked at the hydrodynamics affecting UUS as well as the kinematics and kinetics using a towing mechanism [42, 43] or had the swimmers performing UUS in a swimming flume at different speeds [17, 34, 38, 46]. There was considerable between-study variation in the outcome measures assessed and the data collection and analysis procedures. It is also possible that the variety of UUS methodologies used may have had significant implications in the comparison of results between studies.

#### Study Population

The between study differences in kinematic, kinetic and kinanthropometric characteristics may have also been influenced by differences in sex, age and performance level. The majority of studies reviewed generally consisted of a small sample size and a potentially greater bias towards male compared to female swimmers. Only two of the observational studies consisted entirely of female swimmers, and the seven studies that had a mix of females and males typically had a greater number of male participants compared to females with the exception of two studies [17, 34]. In addition, the majority of studies did not provide very clear descriptions of the level of swimmer, which may be as a result of an inconsistency in the description of different levels of swimmers and lack of standardisation across research. As such, it is difficult to determine how sex, age and performance level may influence UUS velocity and the kinematics and kinetics of this movement.

#### Study Design

The smaller sample sizes seen in the majority of the included studies suggest relatively broad confidence intervals for the outcomes as well as the correlations between outcomes reported in many of the studies. Therefore, there may be some uncertainty regarding what constitutes representative values for different levels of swimmers as well as the strength of correlations between these variables. The small sample sizes may have also contributed to relative lack of statistical power when comparing between groups or conditions, which may have meant some true significant differences were not observed. Further, there is no evidence of any randomised controlled trials conducted in the literature, limiting any understanding of true cause-and-effect relationships between variables.

Across the 25 studies included in this systematic review, there appears to be an absence of long-term monitoring or interventional research that has sought to determine the chronic effect of any biomechanically informed skill acquisition or strength and conditioning intervention. This lack of longitudinal studies is a major limiting factor within this literature.

## Conclusion

The current systematic review has identified that kick frequency [6, 7, 37], kick amplitude [36, 37], vertical toe velocity [10, 15] and knee angular velocity [15, 34] may be the greatest predictors of UUS velocity. Practical implications of how to optimise these parameters for swimmers with different kinanthropometric characteristics are not yet known as an increase in one characteristic, e.g. kick frequency may actually reduce kick amplitude and potentially UUS velocity.

Due to the greater magnitude of water compared to air resistance, the swimmer will lose horizontal velocity after striking the water. It was observed that UUS velocity, kick frequency and amplitude tend to decrease over the 15 m underwater phase with the initiation of the kick recommended to start at the 6 m mark [36, 37]. Additionally, during the underwater phase of any transition from the wall, maintaining kick amplitude and increasing kick frequency, specifically as the swimmer approaches the surface, will achieve optimal swimming velocity and overall underwater performance [35–37]. Despite the advancement of observational studies and development of an understanding of the determinants that impact UUS performance, there appears to be almost a complete lack of intervention research in the biomechanics of UUS. As a result, there is very limited knowledge on how changes in these biomechanical factors interact during performance and why there is variation across different swimmers. Future research should further examine how to optimise the kinematic and kinetic characteristics with respect to the imposed organism constraints [34], as well as the effect of different biomechanically informed skill acquisition and strength and conditioning interventions to improve aspects of UUS performance. Standardisation of testing methods and outcomes related to UUS would also be beneficial.

## Data Availability

The search term strategy used for each database is provided in the methods section of this systematic review. Data sharing is not applicable to this publication as no datasets were produced or analysed during the study.

## Abbreviations

BSFE: Backstroke start with feet emerged
BSFI: Backstroke start with feet immerged
D: Dorsal
DK: Down kick
F: Female
FINA: Fédération internationale de natation
M: Male
MeSH: Medical Subject Headings
P: Prone
PICO: Population, intervention, comparison/control and outcome
PRISMA: Preferred Reporting Items for Systematic Reviews and Meta-analyses
ROM: Range of motion
SD: Standard deviation
UK: Up kick
UUS: Undulatory underwater kick

## References

[1] Hindle BR, Lorimer A, Winwood P, Keogh JWL (2019). The biomechanics and applications of strongman exercises: a systematic review. Sports Med Open.

[2] Domínguez R, Jesús-Sánchez-Oliver A, Cuenca E, Jodra P, FernandesdaSilva S, Mata-Ordóñez F (2017). Nutritional needs in the professional practice of swimming: a review. J Exerc Nutr Biochem.

[3] Shaw G, Boyd KT, Burke LM, Koivisto A (2014). Nutrition for swimming. Int J Sport Nutr Exerc Metab.

[4] Thng S, Pearson S, Keogh JWL (2019). Relationships between dry-land resistance training and swim start performance and effects of such training on the swim start: a systematic review. Sports Med.

[5] Jones J. Dry land strength and power training to enhance swimming in-water turn performance. School of Medical and Health Sciences. Research Online: Edith Cowan University; 2017. p. 63.

[6] Alves F, Lopes P, Veloso A, Martins-Silva A. Influence of body position on dolphin kick kinematics. In: XXIV international symposium on biomechanics in sports. Salzburg, Austria, University of Salzburg: International Society of Biomechanics in Sports; 2006. p. 67–70.

[7] Hochstein S, Blickhan R (2011). Vortex re-capturing and kinematics in human underwater undulatory swimming. Hum Mov Sci.

[8] Takeda T, Sakai S, Takagi H (2020). Underwater flutter kicking causes deceleration in start and turn segments of front crawl. Sports Biomech.

[9] Natation FID: Fina Swimming Rules. 2017. https://resources.fina.org/fina/document/2021/01/12/b3885f9b-630a-4390-861d-4e7f6031f4a4/2017_2021_swimming_16032018.pdf.

[10] Atkison RR, Dickey JP, Dragunas A, Nolte V (2014). Importance of sagittal kick symmetry for underwater dolphin kick performance. Hum Mov Sci.

[11] Cortesi M, Gatta G (2015). Effect of the swimmer's head position on passive drag. J Hum Kinet.

[12] Vennell R, Pease D, Wilson B (2006). Wave drag on human swimmers. J Biomech.

[13] Lyttle A, Blanksby B, Elliott B, Lloyd D. A comparison of underwater gliding and kicking techniques. In: Sanders RHG, editors. XVII international symposium on biomechanics in sports. Edith Cowan University, Perth, Western Australia: Perth School of Biomedical and Sports Science, Edith Cowan University; 1999. p. 81–4.

[14] Elipot M, Dietrich G, Hellard P, Houel N (2010). High-level swimmers' kinetic efficiency during the underwater phase of a grab start. J Appl Biomech.

[15] Higgs AJ, Pease DL, Sanders RH (2017). Relationships between kinematics and undulatory underwater swimming performance. J Sports Sci.

[16] Shimojo H, Sengoku Y, Miyoshi T, Tsubakimoto S, Takagi H (2014). Effect of imposing changes in kick frequency on kinematics during undulatory underwater swimming at maximal effort in male swimmers. Hum Mov Sci.

[17] Hochstein S, Blickhan R (2014). Body movement distribution with respect to swimmer's glide position in human underwater undulatory swimming. Hum Mov Sci.

[18] Sanders RH, Cappaert JM, Devlin RK (1995). Wave characteristics of butterfly swimming. J Biomech.

[19] Gavilán A, Arellano R, Sanders R (2006). Underwater undulatory swimming: study of frequency, amplitude and phase characteristics of the 'body wave'. Port J Sport Sci Suppl Biomech Med Swim X.

[20] McHenry MJ, Pell CA, Long JH (1995). Mechanical control of swimming speed: stiffness and axial wave form in undulating fish models. J Exp Biol.

[21] Hochstein S, Meyer A, Blickhan R. Effect of kick frequency variation on swimming speed and kinematics in human underwater undulatory swimming. In: Exercise JSoSiSaW, editor. Biomechanics and medicine in swimming, XIII. Tokyo: Impress R&D; 2018. p. 71–8.

[22] Pyne DB, Sharp RL (2014). Physical and energy requirements of competitive swimming events. Int J Sport Nutr Exerc Metab.

[23] von Loebbecke A, Mittal R, Fish F, Mark R (2009). A comparison of the kinematics of the dolphin kick in humans and cetaceans. Hum Mov Sci.

[24] Willems TM, Cornelis JA, De Deurwaerder LE, Roelandt F, De Mits S (2014). The effect of ankle muscle strength and flexibility on dolphin kick performance in competitive swimmers. Hum Mov Sci.

[25] Connaboy C, Coleman S, Sanders RH (2009). Hydrodynamics of undulatory underwater swimming: a review. Sports Biomech.

[26] Bingul BM, Tore O, Bulgan C, Aydin M (2015). The kinematic analysis of the grab, rear track and front track start in swimming. Sport Mont.

[27] Slawson S, Conway P, Cossor J, Chakravorti N, West A (2013). The categorisation of swimming start performance with reference to force generation on the main block and footrest components of the Omega OSB11 start blocks. J Sports Sci.

[28] Tor E, Pease DL, Ball KA (2015). Key parameters of the swimming start and their relationship to start performance. J Sports Sci.

[29] Page MJ, McKenzie JE, Bossuyt PM, Boutron I, Hoffmann TC, Mulrow CD (2021). The PRISMA 2020 statement: an updated guideline for reporting systematic reviews. J Clin Epidemiol.

[30] Natera AO, Cardinale M, Keogh JWL (2020). The effect of high volume power training on repeated high-intensity performance and the assessment of repeat power ability: a systematic review. Sports Med.

[31] Hindle BR, Lorimer A, Winwood P, Keogh JWL (2020). A systematic review of the biomechanical research methods used in strongman studies. Sports Biomech.

[32] Davids EL, Roman NV (2014). A systematic review of the relationship between parenting styles and children's physical activity. Afr J Phys Health Educ Recreat Dance.

[33] Arellano R, Gavilan A, Garcia F. A comparison of the underwater undulatory swimming technique in two different body positions. In: VIII international symposium on biomechanics and medicine in swimming. University of Jyvaskyla, Finland: Biomechanics and Medicine in Swimming VIII; 1998. p. 25–8.

[34] Connaboy C, Naemi R, Brown S, Psycharakis S, McCabe C, Coleman S (2016). The key kinematic determinants of undulatory underwater swimming at maximal velocity. J Sports Sci.

[35] de Jesus K, de Jesus K, Machado L, Fernades R, Vilas-Boas JP. Linear kinematic of the underwater undulatory swimming phase performed after two backstroke starting techniques. In: International symposium on biomechanics in sports: conference proceedings archive; 2012. p. 371–4.

[36] Houel N, Elipot M, Andrée F, Hellard H. Kinematics analysis of undulatory underwater swimming during a grab start of national level swimmers. In: XIth international symposium for biomechanics & medicine in swimming. 2010(11), p. 97–9.

[37] Houel N, Elipot M, Andre F, Hellard P (2013). Influence of angles of attack, frequency and kick amplitude on swimmer's horizontal velocity during underwater phase of a grab start. J Appl Biomech.

[38] Shimojo H, Gonjo T, Sakakibara J, Sengoku Y, Sanders R, Takagi H (2019). A quasi three-dimensional visualization of unsteady wake flow in human undulatory swimming. J Biomech.

[39] Wang N, Liu Y. Kinetic chain application to the dolphin kick in butterfly swimming. In: International society of biomechanics in sports, proceedings of XXIV international symposium on biomechanics in sports 2006, Salzburg, Austria, University of Salzburg, c2006; 2006. p. 469–71.

[40] Yamakawa KK, Shimojo H, Takagi H, Tsubakimoto S, Sengoku Y (2017). Effect of increased kick frequency on propelling efficiency and muscular co-activation during underwater dolphin kick. Hum Mov Sci.

[41] Ikeda Y, Ichikawa H, Shimojo H, Nara R, Baba Y, Shimoyama Y (2021). Relationship between dolphin kick movement in humans and velocity during undulatory underwater swimming. J Sports Sci.

[42] Lyttle A, Blanksby B. A look at gliding and underwater kicking in the swim turn. In: Sanders RHY, editor. XVIII international symposium on biomechanics in sports. The Chinese University of Hong Kong, Hong Kong: Applied program: application of biomechanical study in swimming; 2000. p. 56–63.

[43] Lyttle AD, Blanksby BA, Elliott BC, Lloyd DG (2000). Net forces during tethered simulation of underwater streamlined gliding and kicking techniques of the freestyle turn. J Sports Sci.

[44] Hochstein S, Pacholak S, Brücker C, Siebert T, Blickhan R. Drag reduction by underwater undulatory swimming? An experimental and numerical approach. In: XIIth international symposium on biomechanics and medicine in swimming. Canberra: Australian Institute of Sport; 2014. p. 141–7.

[45] Jensen RK, McIlwain J, Terauds J, Bedingfield EW (1979). Modeling lower extremity forces in the dolphin kick. Swimming III, Baltimore.

[46] Miwa T, Matsuuchi K, Shintani H, Kamata E, Nomura T. Unsteady flow measurement of dolphin kicking wake in sagittal plane using 2C-PIV. In: Xth congress of biomechanics and medicine in swimming. Portuguese Journal of Sport Sciences; 2006. p. 64–6.

[47] Shimojo H, Ichikawa H, Tsubakimoto S, Takagi H. The effect of a target sound made by a model swimmer's dolphin kick movement on another swimmer's dolphin kick performance. In: XIth international symposium for biomechanics & medicine in swimming; 2010. p. 341–3.

[48] Shimojo H, Nara R, Baba Y, Ichikawa H, Ikeda Y, Shimoyama Y (2019). Does ankle joint flexibility affect underwater kicking efficiency and three-dimensional kinematics?. J Sports Sci.

[49] Yamakawa KK, Takagi H, Sengoku Y. Three-dimensional analysis of hip and knee joint movements during dolphin kicking and butterfly swimming. In: XIII biomechanics and medicine in swimming. Tokyo: Impress R&D; 2018. p. 187–92.

[50] Arellano R, Pardillo S, Gavilán A. Underwater undulatory swimming: Kinematic characteristics, vortex generation and application during the start, turn and swimming strokes. In: Proceedings of the XXth international symposium on biomechanics in sports. 2002.

[51] Matsuuchi K, Hashizume T, Nakazawa Y, Nomura T, Shintani H, Miwa T (2006). Flow visualization of unsteady flow field around a monofin using PIV. X Biomech Med Swim.

[52] Bressel E (2004). Innovative analyses of human movement: analytical tools for human movement research. Med Sci Sports Exerc.

[53] Connaboy C, Coleman S, Moir G, Sanders R (2010). Measures of reliability in the kinematics of maximal undulatory underwater swimming. Med Sci Sports Exerc.

[54] Castell S, Lighthill J (1976). Mathematical biofluid dynamics. Math Gaz.

[55] Videler J, Kamermans P (1985). Differences between upstroke and downstroke in swimming dolphins. J Exp Biol.

[56] Ungerechts B, Persyn U, Colman V. Analysis of swimming techniques using vortex traces. In: 18 international symposium on biomechanics in sports. Hong Kong: ISBS—conference proceedings archive; 2000.

[57] Cohen RC, Cleary PW, Mason BR (2012). Simulations of dolphin kick swimming using smoothed particle hydrodynamics. Hum Mov Sci.

[58] Nakashima M (2009). Simulation analysis of the effect of trunk undulation on swimming performance in underwater dolphin kick of human. J Biomech Sci Eng.

[59] Tokumaru PT, Dimotakis PE (1991). Rotary oscillation control of a cylinder wake. J Fluid Mech.

[60] Triantafyllou M, Techet A, Zhu Q, Beal D, Hover F, Yue D (2002). Vorticity control in fish-like propulsion and maneuvering. Integr Comp Biol.

[61] Streitlien K, Barrett D, Triantafyllou M (1998). Oscillating foils of high propulsive efficiency. J Fluid Mech.

[62] Jagomägi G, Jürimäe T (2005). The influence of anthropometrical and flexibility parameters on the results of breaststroke swimming. Anthropol Anz.

[63] McCullough AS, Kraemer WJ, Volek JS, Solomon-Hill GF, Hatfield DL, Vingren JL (2009). Factors affecting flutter kicking speed in women who are competitive and recreational swimmers. J Strength Cond Res.

[64] Sugimoto S, Nakashima M, Ichikawa H, Miwa T, Takeda T (2008). The effects of plantar flexion angle increment on the performance during underwater dolphin kick using simulation analysis. Jpn J Phys Educ Health Sport Sci.

[65] Kippenhan B. Lower-extremity joint angles used during the breaststroke whip kick. In: Proceedings of swim sessions: XIX international symposium on biomechanics in sports. San Francisco;2001. p. 48–52.

[66] Zamparo P, Pendergast DR, Termin B, Minetti AE (2002). How fins affect the economy and efficiency of human swimming. J Exp Biol.

[67] Von Loebbecke A, Mittal R, Fish F, Mark R (2009). Propulsive efficiency of the underwater dolphin kick in humans. J Biomech Eng.

[68] Newell KM. Constraints on the development of coordination. In: Wade MG, editor. Motor development in children: aspects of coordination and control. The Netherlands: Martinus Nijhoff, Dordrecht; 1986. p. 341–60.

[69] Elipot M, Hellard P, Taïar R, Boissière E, Rey JL, Lecat S (2009). Analysis of swimmers' velocity during the underwater gliding motion following grab start. J Biomech.

